# Multi-Layer Control with Disturbance Observers for a Long-Travel Dual-Stage Precision Positioning Platform

**DOI:** 10.3390/mi17070773

**Published:** 2026-06-25

**Authors:** Fu-Cheng Wang, Yu-Chi Zane Wang, Yan-Teng Chang, Bo-Xuan Zhong, Yu-Cheng Hsueh, Tien-Tung Chung, Jia-Yush Yen

**Affiliations:** 1 Department of Mechanical Engineering, National Taiwan University, Taipei 10617, Taiwan; zanewang@ntu.edu.tw (Y.-C.Z.W.); r11522855@ntu.edu.tw (Y.-T.C.); r12522846@ntu.edu.tw (B.-X.Z.); r14522823@ntu.edu.tw (Y.-C.H.); ttchung@ntu.edu.tw (T.-T.C.); or jyen@mail.ntust.edu.tw (J.-Y.Y.); 2 Department of Mechanical Engineering, National Taiwan University of Science and Technology, Taipei 10617, Taiwan

**Keywords:** control, disturbance observer, PZT, motor, stage, feedforward, switching, gain-scheduling, fabrication

## Abstract

This paper investigates the effects of disturbance observers on a long-travel precision positioning platform. We propose a multi-layer control architecture, including a disturbance observer, a feedforward compensator, gain-scheduling, and control switching. The platform consists of motor and piezoelectric transducer (PZT) stages to enable nanometre-level accuracy within 10 cm travel ranges. We identified the dynamic models of the stages through experiments and applied them to develop control designs. The PZT stage was equipped with feedforward compensators, a disturbance observer and real-time switching control schemes to achieve robust and precise tracking. On the other hand, we applied gain-scheduling and feedforward compensation to the motor stages to track large displacements. The control effects of the integrated platform were validated through simulations and experiments and demonstrated significant improvements in accuracy and robustness. Finally, the platform was incorporated with two-photon polymerisation to fabricate micro-lenses. This work evaluates the lenses’ optical properties to highlight the advantages provided by the multiple control structure for improving precision and microfabrication applications.

## 1. Introduction

Recent advancements in technologies have enabled the continued miniaturisation of components and systems, with a resulting increase in demands for high-precision systems and extensive research on precision motion systems. This paper presents a multi-layer control structure, consisting of piezoelectric transducer (PZT) and motor stages, that is designed to enhance the performance of a long-travel precision platform.

PZTs are widely adopted in precision applications because of their inherent merits, which include a high resolution and rapid dynamic responses. For example, Williams et al. [1] proposed low-cost and lightweight piezoelectric actuators to suppress structural vibrations. However, the nonlinear properties of PZTs, such as hysteresis and creep, can significantly degrade positioning accuracy if left uncompensated. To address these issues, numerous mathematical models have been proposed to characterise PZT nonlinearities. For instance, Delibas et al. [2] introduced a micromechanical model to capture the rate-dependent behaviour of piezoelectric materials and reproduce frequency-dependent hysteresis characteristics. Gu and Zhu [3] proposed an analytical ellipse-based model to describe rate-dependent hysteresis in piezoelectric actuators. Gan and Zhang [4] proposed a Bouc–Wen hysteresis model and applied least square methods to identify model parameters. Chen et al. [5] applied a modified Bouc–Wen model to capture asymmetric hysteresis behaviours, whereas Fang et al. [6] utilised particle swarm optimisation (PSO) to identify frequency-dependent Bouc–Wen model parameters. Wang et al. [7] developed an asymmetric Prandtl–Ishlinskii model to represent complex hysteresis loops. Baziyad et al. [8] developed a hysteresis-modelling and compensation framework that integrated Preisach hysteresis operators with support vector machines and PSO to describe the dynamic behaviours of piezoelectric actuators.

Based on these PZT models, researchers have employed control techniques to enhance PZT performance. For instance, Wang et al. [7] developed an inverse Prandtl–Ishlinskii-based hysteresis compensation method, whereas Baziyad et al. [8] applied a feedforward compensation scheme combined with proportional–integral–derivative control to Preisach-type systems. Shen and Homaifar [9] developed and experimentally validated several multivariable control strategies for suppressing resonance vibrations in flexible structures employing PZT actuators. Wang et al. [10] designed an adaptive integral control for a PZT stage to enable the adjustment of control gains according to positioning errors.

Engineering systems often exhibit multiple, and sometimes conflicting, performance requirements, such as fast transient responses, minimal overshoot, and smooth steady-state behaviour. To meet these demands, various switching control frameworks have been developed. For example, Qin et al. [11] proposed a fuzzy adaptive PID controller for fuel cell systems, while Xu et al. [12] introduced a fuzzy PID approach for marine vessels. Asl et al. [13] developed a fuzzy switching controller that integrates PID control with linear quadratic regulation for robotic applications. More recently, Li et al. [14] proposed a switching mode control strategy for motor systems, and Artetxe et al. [15] combined feedforward neural networks with sliding mode control to improve the tracking accuracy of a PZT actuator. In addition, Sivrioglu [16] presented a multi-objective control approach to suppress vibrations in a flexible blade subject to uncertain PZT actuation forces.

A control switching mechanism was proposed for a PZT stage [17], in which two predefined robust controllers were selected based on predicted system responses. This approach was integrated into a two-photon polymerization (TPP) system for microstructure fabrication, where it produced micro-lenses with a higher intensity and improved sharpness compared to a fixed controller. Subsequently, Wang et al. [18] introduced a multiple switching control structure, which further enhanced the sharpness of the fabricated micro-lenses by 35%. Model estimation techniques were later incorporated to update system parameters within the prediction mechanism, thereby improving forecasting accuracy and overall system performance [19,20]. These frameworks achieved up to a 43% improvement in the image sharpness of the fabricated micro-lenses [20]. Building on these developments, the present paper further advances the approach by integrating a feedforward compensator and a disturbance observer (DOB) to enhance positioning accuracy and micro-lens quality.

While PZT stages can achieve nanometre-level positioning precision, their applicability is restricted by their inherently limited travel range. A common solution is to integrate PZT stages with long-stroke motion stages to simultaneously achieve both a large travel range and high resolution. In this work, a PZT stage is combined with motor stages to extend the travel range while maintaining precision motion. We apply feedforward and gain-scheduling controls to the motor stages to enhance their tracking accuracy and dynamic performance. The feedforward control can compensate for phase lags, while gain-scheduling can adjust control magnitudes according to real-time errors.

Finally, the hybrid platform is applied for microstructure fabrication using a TPP system. TPP is a nonlinear optical lithography method with two-photon absorption mechanisms, which enable both localised polymerisation within photosensitive materials and microfabrication [21,22,23]. By integrating the platform with a TPP system, this work demonstrates how precision engineering techniques can be synergistically combined with advanced manufacturing processes to enable interdisciplinary applications in microfabrication. Finally, we fabricated micro-lenses and analysed their optical performance to validate the system’s effectiveness.

The remainder of this paper is organised as follows. Section 2 introduces the long-stroke precision platform. Section 3 presents the control strategies for the PZT stage. Section 4 depicts the control methods for the motor stages. Section 5 assesses the platform performance by simulations and experiments. Section 6 applies the combined system to micro-lens fabrication via TPP, along with an optical performance assessment. Finally, Section 7 provides concluding remarks.

## 2. The Long-Travel Precision Platform

**Figure 1:**
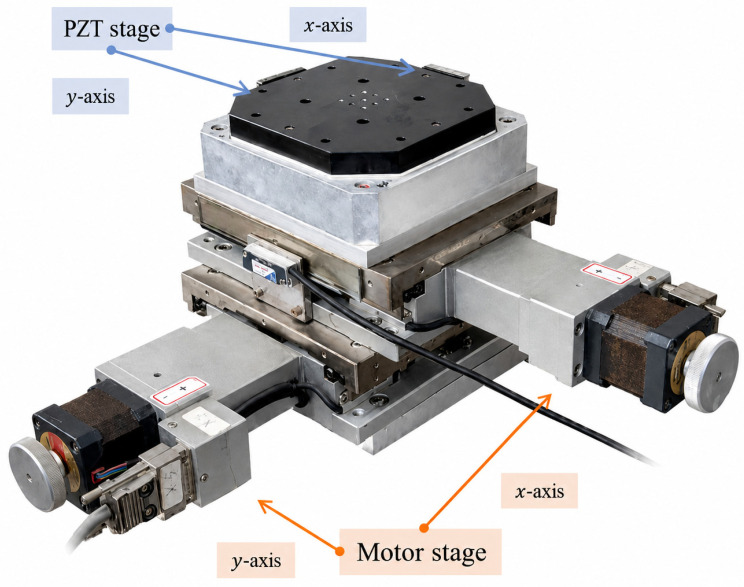
The long-travel precision platform.

Figure 1 illustrates a platform that integrates the PZT and motor stages to achieve long travel with precision accuracy. The PZT stage has encoders featuring resolutions of 1.22 nm within 100 μm [24]. Complementarily, the motor stages have strokes of 100 mm and encoders with resolutions of 0.1 μm [25,26]. Detailed stage specifications are provided in Appendix A. This combined configuration enables nanometre-precision positioning across centimetre-scale motion ranges.

As the stages are commercial products, detailed system characteristics and specifications are not fully disclosed. Therefore, system identification experiments were conducted to obtain the stage models. During these experiments, a sample holder for TPP fabrication was integrated with the stage. We applied swept-sine excitations and measured the corresponding displacement responses [17]. Due to inherent system uncertainties, the identification processes were repeated ten times for both the *x*- and *y*-axes, yielding 10 models for each axis.

The identified PZT stage models are as follows:

(1) $$\begin{array}{l} G_{P}^{x_{1}}=\frac{798.3}{s+95.60},\;G_{P}^{x_{2}}=\frac{797.2}{s+95.65},\;G_{P}^{x_{3}}=\frac{801.2}{s+96.34},\;G_{P}^{x_{4}}=\frac{801.4}{s+96.35},\\ G_{P}^{x_{5}}=\frac{801.5}{s+96.51},\;G_{P}^{x_{6}}=\frac{800.0}{s+96.40},\;G_{P}^{x_{7}}=\frac{799.5}{s+96.44},\;G_{P}^{x_{8}}=\frac{802.2}{s+96.87},\\ G_{P}^{x_{9}}=\frac{800.7}{s+96.70},\;G_{P}^{x_{10}}=\frac{801.5}{s+96.88},\;G_{P}^{y_{1}}=\frac{1325.0}{s+200.60},\;G_{P}^{y_{2}}=\frac{1321.0}{s+200.40},\\ G_{P}^{y_{3}}=\frac{1326.0}{s+201.60},\;G_{P}^{y_{4}}=\frac{1331.0}{s+203.00},\;G_{P}^{y_{5}}=\frac{1319.0}{s+200.90},\;G_{P}^{y_{6}}=\frac{1326.0}{s+202.30},\\ G_{P}^{y_{7}}=\frac{1325.0}{s+202.30},\;G_{P}^{y_{8}}=\frac{1321.0}{s+201.60},\;G_{P}^{y_{9}}=\frac{1328.0}{s+202.60},\;G_{P}^{y_{10}}=\frac{1322.0}{s+202.50}, \end{array}$$

where the subscript *_P_* denotes the PZT stage, while the superscripts _*x*_*i*__ and _*y*_*i*__ indicate the *i*-th model for respective axes.

Similarly, the following ten motor-stage models were obtained for each axis:

(2) $$\begin{array}{l} G_{M}^{x_{1}}=\frac{0.1004}{s},\;G_{M}^{x_{2}}=\frac{0.1013}{s},\;G_{M}^{x_{3}}=\frac{0.0998}{s},\;G_{M}^{x_{4}}=\frac{0.1015}{s},\;\\ G_{M}^{x_{5}}=\frac{0.1007}{s},\;G_{M}^{x_{6}}=\frac{0.1009}{s},\;G_{M}^{x_{7}}=\frac{0.1017}{s},\;G_{M}^{x_{8}}=\frac{0.1010}{s},\\ G_{M}^{x_{9}}=\frac{0.1012}{s},\;G_{M}^{x_{10}}=\frac{0.1011}{s},\;G_{M}^{y_{1}}=\frac{0.1011}{s},\;G_{M}^{y_{2}}=\frac{0.1014}{s},\\ G_{M}^{y_{3}}=\frac{0.1005}{s},\;G_{M}^{y_{4}}=\frac{0.9999}{s},\;G_{M}^{y_{5}}=\frac{0.1010}{s},\;G_{M}^{y_{6}}=\frac{0.1015}{s},\\ G_{M}^{y_{7}}=\frac{0.1002}{s},\;G_{M}^{y_{8}}=\frac{0.1013}{s},\;G_{M}^{y_{9}}=\frac{0.1019}{s},\;G_{M}^{y_{10}}=\frac{0.0994}{s}, \end{array}$$

where the subscript *_M_* represents the motor stage.

The gap metric [27] was employed to select suitable nominal models for control design, as it quantifies the robustness margin between plants. Assume that a nominal plant $G_{0}={\tilde{M}}^{-1}\;\tilde{N}$ and a perturbed plant $G_{\Delta }={\left(\tilde{M}+\Delta_{\tilde{M}}\right)}^{-1}(\tilde{N}+\Delta_{\tilde{N}})$, where Δ $\tilde{M}{\tilde{M}}^{*}+\tilde{N}{\tilde{N}}^{*}=I\;\mathrm{and}\;\Delta_{\tilde{M}},\;\Delta_{\tilde{N}}\in RH_{\infty }$; the gap between $G_{0}$ and $G_{\Delta }$ is then defined as $\delta (G_{0},\;G_{\Delta })$, which represents the minimum allowable perturbation $\Delta =\left[\begin{array}{cc} \Delta_{\tilde{N}} & -\Delta_{\tilde{M}} \end{array}\right]$ that can transfer $G_{0}$ into $G_{\Delta }$ [27]. The nominal model is selected to minimise the worst-case gap over all identified candidates.

Based on this analysis, the selected nominal plants are as follows:

(3) $$G_{P}^{x}=\frac{797.2}{s+96.65},\;G_{P}^{y}=\frac{1325.0}{s+202.30},\;G_{M}^{x}=\frac{0.1015}{s},\;G_{M}^{y}=\frac{0.1010}{s},\;$$

representing the nominal dynamics of the PZT and motor stages along each axis.

## 3. Control Designs for the PZT Stage

**Figure 2:**
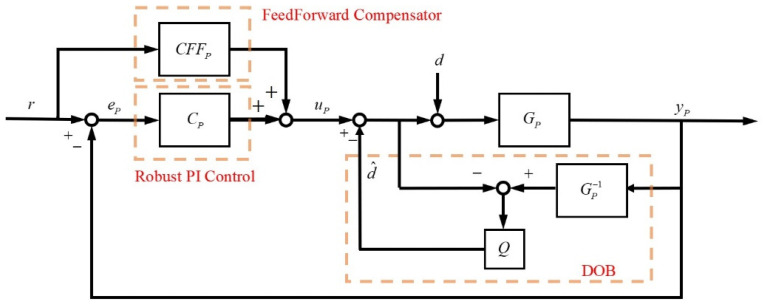
Control strategies for the PZT stage.

This section presents the controls for the PZT stage, shown in Figure 2, where $G=G_{P}^{x}\;\mathrm{or}\;G_{P}^{y}$. The proposed architecture integrates a robust proportional–integral (PI) controller $C_{P}$, a DOB, and a feedforward compensator $CFF_{P}$. The robust PI controller provides a simple yet effective structure for achieving system robustness. The DOB enhances the disturbance rejection capability, while the feedforward compensator mitigates phase lag to improve the overall dynamic response.

### 3.1. Robust PI Control Design

**Figure 3:**
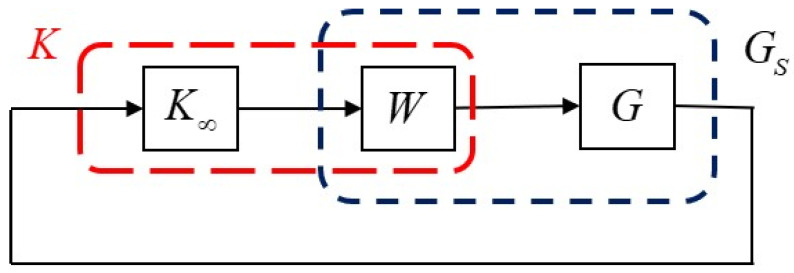
Loop-shaping.

The robust controller was designed using loop-shaping techniques [28,29,30], as shown in Figure 3. A weighting function *W* is first applied to shape the plant as $G_{S}=GW$, from which a controller $K_{\infty }$ is synthesised. The resulting weighted controller $K=WK_{\infty }$ is then implemented on *G*.

Considering multiple system requirements, we selected the following weighting functions

(4) $$\begin{array}{l} W_{f}^{x}=\frac{40(s+50\pi )}{s(s+40\pi )},\;W_{m}^{x}=\frac{18(s+20\pi )}{s(s+35\pi )},\;W_{s}^{x}=\frac{12.5(s+10\pi )}{s(s+30\pi )},\;\\ W_{f}^{y}=\frac{50(s+50\pi )}{s(s+40\pi )},\;W_{m}^{y}=\frac{18(s+20\pi )}{s(s+35\pi )},\;W_{s}^{y}=\frac{12.5(s+15\pi )}{s(s+30\pi )},\; \end{array}$$

and designed the following robust controllers [31]:

(5) $$\begin{array}{l} K_{f}^{x}=\frac{6.144\times 10^{6}s^{3}+2.594\times 10^{9}s^{2}+3.683\times 10^{11}s+1.765\times 10^{13}}{s^{5}+8.288\times 10^{4}s^{4}+5.663\times 10^{7}s^{3}+1.106\times 10^{10}s^{2}+6.596\times 10^{11}s},\;\\ K_{m}^{x}=\frac{7.929\times 10^{5}s^{3}+2.192\times 10^{8}s^{2}+1.900\times 10^{10}s+5.251\times 10^{11}}{s^{5}+3.399\times 10^{4}s^{4}+1.541\times 10^{7}s^{3}+1.894\times 10^{9}s^{2}+6.707\times 10^{10}s},\\ K_{s}^{x}=\frac{2.590\times 10^{5}s^{3}+5.683\times 10^{7}s^{2}+3.491\times 10^{9}s+6.160\times 10^{10}}{s^{5}+1.996\times 10^{4}s^{4}+7.377\times 10^{6}s^{3}+6.810\times 10^{8}s^{2}+1.564\times 10^{10}s},\\ K_{f}^{y}=\frac{8.492\times 10^{6}s^{3}+4.519\times 10^{9}s^{2}+7.836\times 10^{11}s+4.450\times 10^{13}}{s^{5}+1.026\times 10^{5}s^{4}+8.927\times 10^{7}s^{3}+1.905\times 10^{10}s^{2}+1.188\times 10^{12}s},\\ K_{m}^{y}=\frac{7.199\times 10^{5}s^{3}+2.616\times 10^{8}s^{2}+2.650\times 10^{10}s+8.111\times 10^{11}}{s^{5}+3.543\times 10^{4}s^{4}+1.893\times 10^{7}s^{3}+2.340\times 10^{9}s^{2}+8.197\times 10^{10}s},\\ K_{s}^{x}=\frac{2.456\times 10^{5}s^{3}+8.737\times 10^{7}s^{2}+8.460\times 10^{9}s+2.303\times 10^{11}}{s^{5}+2.039\times 10^{4}s^{4}+1.024\times 10^{6}s^{3}+1.268\times 10^{9}s^{2}+4.250\times 10^{10}s}. \end{array}$$

The superscripts ^*x*, *y*^ denote the *x*- and *y*-axis, respectively, while the subscripts _*f*, *m*, *s*_ indicate fast, medium, and smooth controllers, respectively. The fast controls provide rapid but oscillatory responses, as shown in Figure 3, whereas the smooth controllers yield smooth yet highly damped dynamics. The medium controllers offer a balance between these two behaviours.

Because these controllers are fifth-order and thus unsuitable for real-time implementation, PSO algorithms [32] were employed to reduce them to the following PI controls:

(6) $$\begin{array}{l} C_{f}^{x}=0.085+\frac{32.827}{s},\;C_{m}^{x}=0.073+\frac{7.856}{s},\;C_{s}^{x}=0.027+\frac{4.732}{s},\\ C_{f}^{x}=0.071+\frac{43.958}{s},\;C_{m}^{x}=0.034+\frac{10.375}{s},\;C_{s}^{x}=0.049+\frac{5.759}{s}. \end{array}$$

**Figure 4:**
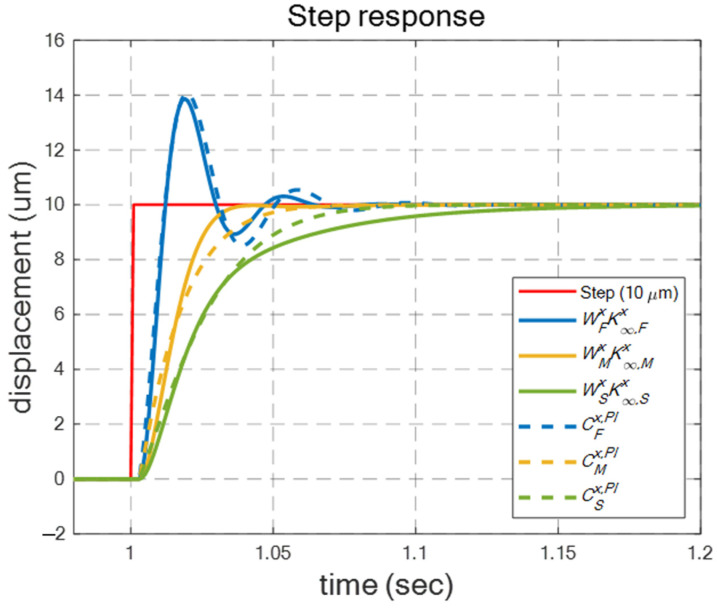
Step responses of the PZT stage.

The step responses employing the high-order and PI controllers are compared in Figure 4. While fast controllers maintain quick but oscillatory responses and smooth controllers remain slower and overdamped, the robust PI controllers achieve nearly identical performance with substantially reduced structural complexity. Hence, the robust PI controllers were implemented for both simulations and experiments.

### 3.2. Feedforward Compensator

From the system structure in Figure 2,

(7) $$\begin{array}{l} y_{P}=G_{P}\cdot u_{P}\\ \;\;\;\;=G_{P}\cdot (CFF_{P}\cdot r+C_{P}\cdot (r-y_{P}))\\ \;\;\;\;={\left(I+G_{P}C_{P}\right)}^{-1}(G_{P}\cdot CFF_{P}+G_{P}C_{P})\cdot r. \end{array}$$

A feedforward compensator can be designed as $CFF_{P}=G_{P}^{-1}$ to counteract the phase lag by letting $y_{P}=r$. To ensure controller properness, we added an additional pole, so that

(8) $$CFF_{P}^{x}=\frac{s+96.65}{0.001s+797.2},\;CFF_{P}^{y}=\frac{s+202.30}{0.001s+1325.0}.$$

### 3.3. Disturbance Observer

A DOB was incorporated to further enhance system robustness. In precision positioning systems, disturbances might degrade performance and may even cause instability. As shown in Figure 2, the DOB estimates disturbances using the system input $u_{P}$ and output $y_{P}$, where $y_{P}=G_{P}(u_{P}+\hat{d}+d)$, and the estimated disturbance $\hat{d}$ becomes

$$\hat{d}=Q(G_{P}^{-1}y_{P}-u_{P}-\hat{d})=Qd,$$

where *d* denotes the system disturbance, and *Q* is a low-pass filter, so that $\hat{d}=Qd\approx d$. The estimated disturbance is injected into the inner loop to counteract the disturbance in real time. Finally, experimental evaluations were carried out to determine the low-pass filter *Q* as follows:

(9) $$Q=\frac{60\pi }{s+60\pi }$$

which yielded the best performance with a cutoff frequency of 30 Hz.

### 3.4. Multiple Switching Control

**Figure 5:**
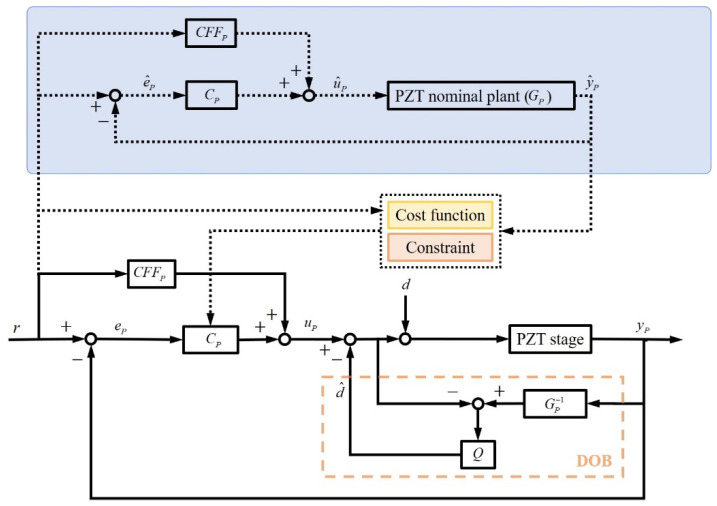
Multiple control switching mechanism for the PZT stage. (dashed arrows indicate signals in the response prediction and control switching).

Practical systems often have multiple performance objectives, such as a fast response, small overshoot, and high tracking accuracy, which cannot be simultaneously satisfied by a fixed controller. Thus, we applied a switching control strategy, shown in Figure 5, which predicts system responses over a future horizon $H_{P}$ and evaluates all possible switching sequences over $S_{P}$ steps. With the three candidate controllers in Equation (6), $3^{S_{P}}$ sequences are possible. The effects of each sequence were evaluated as follows:

(10) $$J={\left(\frac{1}{H_{P}}\cdot \sum_{k}^{k+H_{P}}{\left(r(k)-\hat{y}(k)\right)}^{2}\right)}^{1/2}$$

where $\hat{y}={\hat{y}}_{P}$ when applying the PZT stage alone. Equation (10) represents the root mean square error (RMSE). The sequence that minimises *J* is selected and applied to the system.

### 3.5. Simulation and Experimental Verifications for the PZT Stage

Both simulations and experiments were performed to assess the proposed control architecture. In the simulations, white noise disturbances were added to evaluate the effects of the DOB.

The step responses employing different control strategies are compared in Table 1. With the DOB, performance was improved in all simulations and experiments. In the experiments, the overshoot, RMSE and settling time also improved, although the rise time was slightly degraded, likely due to mechanical friction.

**Figure 6:**
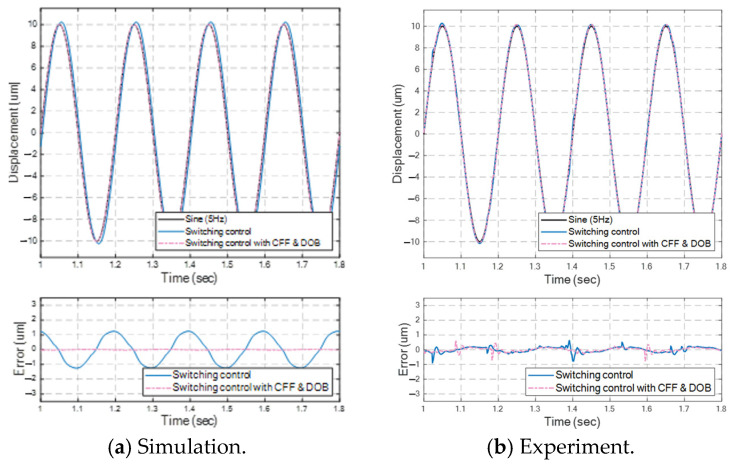
PZT stage responses to sinusoidal inputs.

The results for sinusoidal tracking are provided in Figure 6 and Table 2, which show that adding either a feedforward compensator or DOB enhances all tracking performances in the simulations and experiments. Specifically, significant reductions in the maximum absolute error (MAE) and RMSE were observed. Finally, combining the feedforward compensator and DOB yielded the most significant improvements.

## 4. Control Designs for Motor Stages

**Figure 7:**
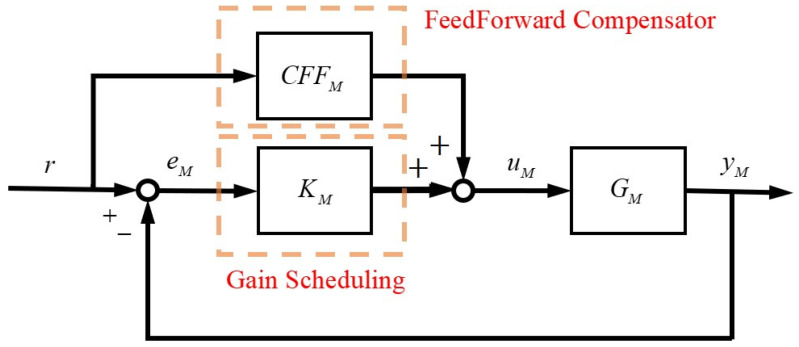
Control strategies for the motor stages.

We applied gain-scheduling control and feedforward compensation techniques to the motor stages, as illustrated in Figure 7, to enhance their dynamic performance. Because the stage models are first-order (see Equation (3)), the closed-loop pole location can be explicitly assigned by tuning the proportional gain $K_{M}$. In addition, a feedforward compensator was incorporated to counteract the inherent phase lag, thereby improving the tracking accuracy.

### 4.1. Gain-Scheduling

The controller gain $K_{M}$ is adjusted according to the tracking error *e*, as follows:

(11) $$K_{M}=\left\{\begin{array}{l} 1600,\;\mathrm{if}\;\left|e_{M}\right|>50\;\mu m,\\ 30(\left|e_{M}\right|-10+400,\;\mathrm{if}\;10\;\mu m\leq \left|e_{M}\right|\leq 50\;\mu m),\\ 400,\;\mathrm{if}\;\left|e_{M}\right|<10\;\mu m. \end{array}\right.$$

This gain-scheduling mechanism enables automatic modification of the control effort under different operating conditions, thereby improving responsiveness when large errors occur, while maintaining stability when the system approaches the reference.

### 4.2. Feedforward Compensation

Because the motor stages exhibit a considerable phase lag, a feedforward compensator with $CFF_{M}=G_{M}^{-1}$ was applied to provide phase-lead compensation. To ensure properness, an additional pole was added, resulting in the following implementations for the *x*- and *y*-axes:

(12) $$CFF_{M}^{x}=\frac{s}{0.0001s+0.1015},\;CFF_{M}^{y}=\frac{s}{0.0001s+0.1010}.$$

The compensator can effectively reduce the phase lag and improve the tracking capability of the motor stages, particularly in high-frequency operations.

### 4.3. Simulation and Experimental Verifications for the Motor Stage

The tracking performance was evaluated using ramp and sinusoidal inputs. Table 3 summarises the results, where the inclusion of the feedforward compensator significantly decreased tracking errors and improved phase characteristics. In particular, the phase lag was largely eliminated and substantial reductions in the MAE and RMSE were observed.

## 5. Integrated Long-Stroke Precision Platform

**Figure 8:**
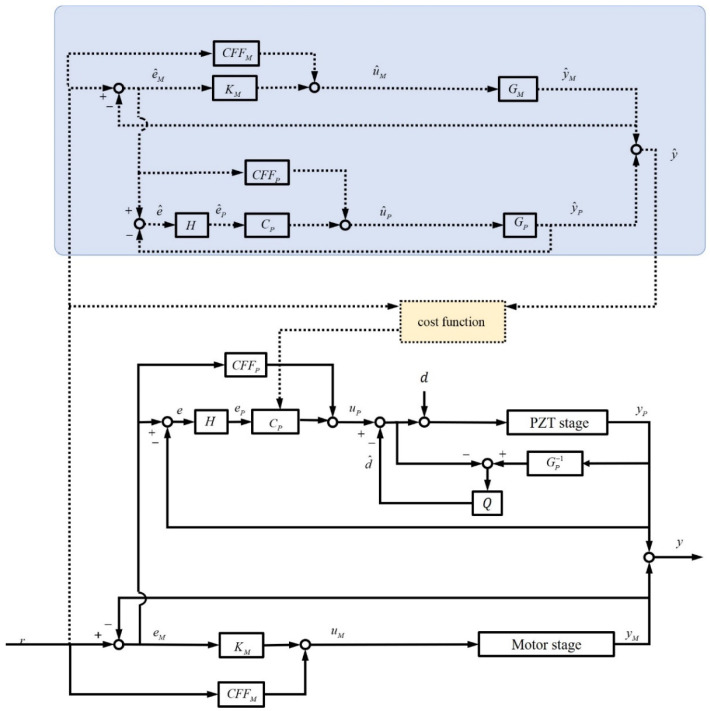
Multiple control structure of the long-stroke precision platform. (dashed arrows indicate signals in the response prediction and control switching).

The PZT and motor stages were integrated to form a long-travel precision platform. A multiple control architecture, illustrated in Figure 8, was implemented to exploit the complementary characteristics of the two stages. During long-range motion, the motor stages provide coarse positioning and accommodate large-displacement and high-speed operation. As the tracking error decreases, a gain-scheduling strategy is applied to gradually reduce the motor velocity, while the PZT stage compensates the residual errors to achieve high precision. This cooperative mechanism enables the system to realise both rapid long-stroke motion and nanometre-level accuracy.

For the PZT stage, disturbance effects were attenuated through the DOB, and the phase lag was mitigated using a feedforward compensator $CFF_{P}$. A response predictor further estimated the future system behaviour, enabling the multiple switching controller to select the optimal control sequence to enhance dynamic performance. For the motor stages, the proportional gain $K_{M}$ was adaptively adjusted based on the tracking error, and a feedforward compensator $CFF_{M}$ was incorporated to eliminate phase lags and improve tracking abilities.

Considering the limited travel range of the PZT stage, a constraint function *H* was introduced to prevent actuator saturation and ensure safe operation, as follows:

(13) $$e_{p}=H(e)=\left\{\begin{array}{l} e,\;\mathrm{if}\;\left|e\right|\leq 50\;\mu m,\\ 0,\;\mathrm{if}\;\left|e\right|>50\;\mu m, \end{array}\right.$$

where $e=r-y_{M}-y_{P}$.

The integrated control architecture was experimentally validated using three types of inputs: step, ramp and sinusoidal signals. Table 4 summarises the results, where the integrated system with the DOB and switching control improved the response time and reduced tracking errors when compared with the motor stages alone.

For sinusoidal tracking, the motor stages alone exhibited noticeable amplitude attenuation and phase lag. The PZT stage compensated the tracking errors, resulting in substantial reductions in the MAE and RMSE, along with the elimination of phase lags. These results confirm that the integrated configuration effectively suppresses tracking errors and provides superior performance for dynamic tracking tasks.

## 6. Fresnel Zone Plate Fabrication

**Figure 9:**
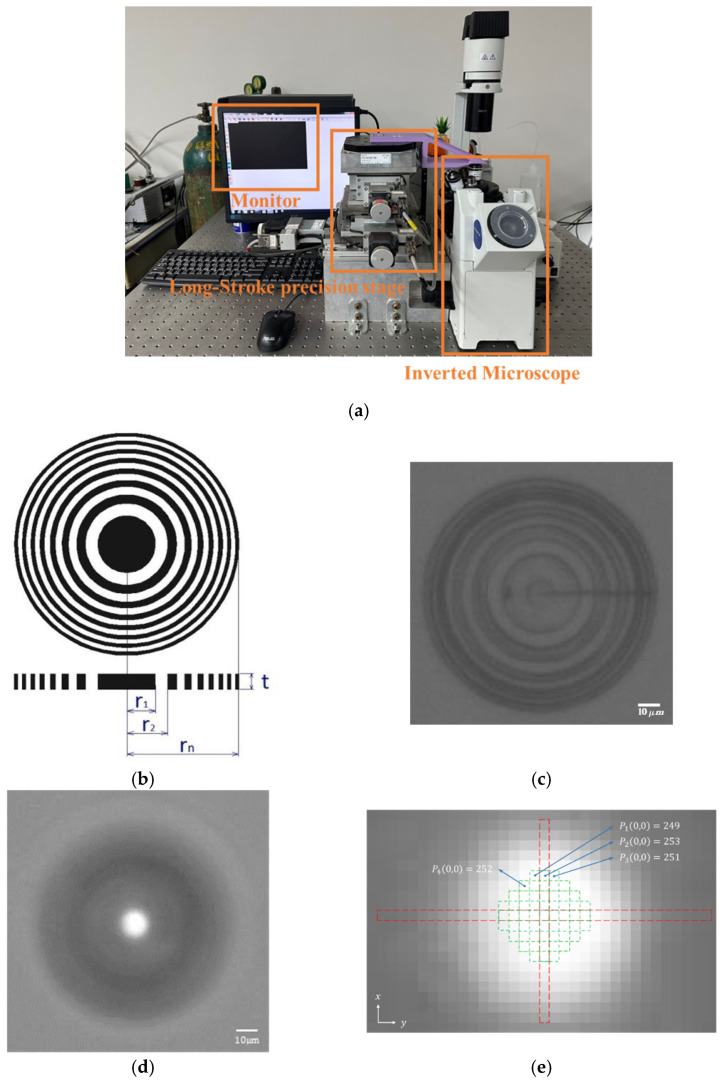

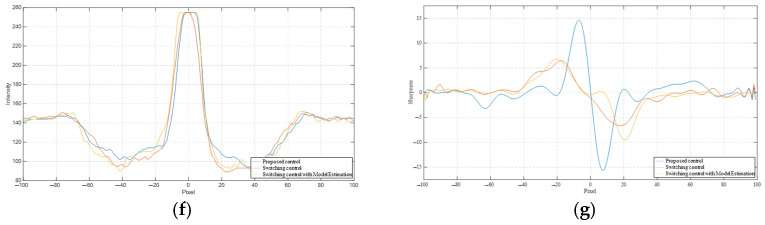
Micro-lens fabrication and analyses. (**a**) The integrated system. (**b**) The FZP design. (**c**) The fabricated FZP. (**d**) The light image on the focal plane. (**e**) Pixels for intensity estimation. (**f**) The intensity curves. (**g**) The sharpness curves.

We applied the hybrid platform to fabricate micro-lenses using a TPP system. TPP is widely employed in micro-photonics [33,34] and electromechanical system fabrication [35,36] because of its capacity to produce high-resolution three-dimensional structures. As shown in Figure 9a, the precision platform was integrated with a TPP system to provide accurate laser positioning during fabrication. An adaptor mounted on the PZT stage held the substrate, enabling the selective curing of the photoresist on a microscope slide by the femtosecond laser.

To demonstrate planar micro-lens focusing, a Fresnel Zone Plate (FZP) with a diameter of 128 μm was fabricated following the procedures described by Wang et al. [18] The radius of the Fresnel zone is given by the following:

(14) $$r_{n}={\left(nf\lambda +\frac{1}{4}n^{2}\lambda^{2}\right)}^{1/2},$$

where $r_{n}$ denotes the radius of the *n*-th zone (see Figure 9b), while *f* and *λ* are the focal length and the wavelength, respectively. As a demonstration, we set *n* = 9, *f* = 500 μm, and *λ* = 632.8 nm to fabricate FZPs. The tracking errors obtained under different control strategies are compared in Table 5. The proposed multiple-control structure achieved superior performance over previously reported methods.

A CMOS camera was used to image the fabricated FZP, as shown in Figure 9c. We evaluated both the light intensity and boundary sharpness to quantify the optical performance and fabrication quality. The focusing behaviour was characterised by capturing the intensity distribution at the focal plane, as shown in Figure 9d, where the brightness of each pixel is represented on a 0–255 grayscale.

To estimate the light intensity, we analysed the brightness values of pixels around the centre of the focused spot (see Figure 9e) and defined the intensity as the average grayscale value (from 0 to 255) of 57 central pixels, expressed as

(15) $$I=\frac{1}{57}\sum_{i=1}^{57}P_{i},$$

where $P_{i}$ is the brightness of pixel *i*.

The light sharpness was evaluated by sampling the grayscale values along the diameter of the focal spot, as shown in Figure 9f. A fitted curve was generated using MATLAB R2024a functions *polyfit* and *polyval*. The sharpness was then defined as the magnitude of the derivative of this fitted curve, as illustrated in Figure 9g. Table 5 summarises the optical quality metrics of FZPs fabricated with different control strategies. These results show that the proposed multi-control architecture achieved the best performance, surpassing the multiple switching control and the model estimation-based switching control.

## 7. Conclusions

This paper demonstrated the advantages of a DOB for a long-travel precision positioning platform. We proposed a control framework for a long-travel precision platform composed of PZT and motor stages. First, implementing a switching control strategy, augmented with feedforward control and a DOB, on the PZT stage enhanced high-precision positioning and suppressed external disturbances. Second, the incorporation of gain-scheduling and feedforward control into the motor stages achieved long-range motion with improved dynamic responses. By combining these mechanisms, the proposed architecture enables the integration of long-stroke travel and nanometre-level precision.

We evaluated the effectiveness of the integrated control structure through both simulations and experiments, which demonstrated significant improvements in tracking accuracy and overall motion performance. We further validated the practical applicability of the system by using the integrated platform to fabricate micro-lenses using TPP. Optical characterisations of the fabricated FZPs confirmed that the enhanced motion control improved the lens quality. Overall, the presented control framework provides a robust and scalable solution, particularly for applications such as microfabrication and photonics, which require both extended travel and high-precision actuation.

## Figures and Tables

**Table 1:** PZT stage tracking performance (step input).

		C.S.	C.S. + DOB	Imp. (%)
Simulation	Overshoot (%)	0.9140	0.8910	5.31
	RMSE (μm)	1.8658	1.6285	27.41
	Settling time (s)	0.0337	0.0280	16.91
	Rising time (s)	0.0191	0.0123	35.60
Experiment	Overshoot (%)	2.6000	1.8200	30.00
	RMSE (μm)	1.8582	1.7122	7.86
	Settling time (s)	0.0480	0.0290	39.58
	Rising time (s)	0.0187	0.0178	4.81

C.S.: control switching; DOB: disturbance observer.

**Table 2:** PZT stage tracking performance (sinusoidal inputs).

			C.S.	C.S. + CFF	C.S. + DOB	C.S. + CFF + DOB
Sinusoidal 1 Hz	Simulation	Phase lag (deg)	3.6000	0	2.5200	0
		MAE (μm)	0.9568	0.0036	0.9518	0.0025
		RMSE (μm)	0.4590	0.0221	0.4511	0.0009
	Experiment	Phase lag (deg)	0.7200	0	0.3600	0
		MAE (μm)	0.3135	0.1635	0.3088	0.1522
		RMSE (μm)	0.1559	0.0614	0.1557	0.0216
Sinusoidal 5 Hz	Simulation	Phase lag (deg)	10.0800	0	9.0000	0
		MAE (μm)	4.5916	0.0981	4.4177	0.0257
		RMSE (μm)	2.5256	0.1277	2.3507	0.0178
	Experiment	Phase lag (deg)	7.2000	−1.8000	5.4000	0
		MAE (μm)	1.5461	0.6868	1.5011	0.7184
		RMSE (μm)	0.8618	0.3290	0.8446	0.1417

C.S.: control switching; CFF: feedforward compensation; DOB: disturbance observer.

**Table 3:** Motor stage tracking performance.

**Ramp Input (100 μm/s)**		**GS**	**GS + CFF**
Simulation	MAE (μm)	2.4631	0.2312
	RMSE (μm)	1.9877	0.0322
Experiment	MAE (μm)	2.7000	0.8000
	RMSE (μm)	2.0131	0.1637
**S** **inusoidal** **input (5 Hz)**		**GS**	**GS + CFF**
Simulation	Phase lag (deg)	28.8000	1.8000
	MAE (μm)	16.8263	2.4438
	RMSE (μm)	12.5136	1.7282
Experiment	Phase lag (deg)	27.0000	5.4000
	MAE (μm)	17.0691	5.9123
	RMSE (μm)	12.7363	3.1630

GS: gain-scheduling; CFF: feedforward compensation.

**Table 4:** Tracking performance of the integrated platform (experiments).

		*x*-Axis		*y*-Axis	
		Motor Stage	Combined Stage	Motor Stage	Combined Stage
Step	Rising time (s)	0.1319	0.1284	0.1181	0.1167
	Settling time (s)	0.1561	0.1526	0.1368	0.1347
	RMSE (μm)	36.9231	36.9102	34.9779	34.9739
Ramp	MAE (μm)	3.3000	1.7740	4.4000	1.9220
	RMSE (μm)	0.3798	0.1868	0.3601	0.2184
Sinusoidal	MAE (μm)	6.7586	2.4382	5.7122	2.4653
	Phase lag (deg)	3.6000	0	2.7000	0
	RMSE (μm)	3.6501	0.8728	4.3508	1.3744

**Table 5:** Performance of FZP fabrication and micro-lenses.

Tracking Errors	C.S.	C.S. + M.E.	Proposed Control
x-axial RMSE (nm)	94.2	92.5	87.1
y-axial RMSE (nm)	113.4	107.8	101.8
Optical properties	C.S.	C.S. + M.E.	proposed control
Light intensity	247.6140	254.2456	254.4516
Sharpness	6.4483	9.5368	15.6208

C.S.: control switching; M.E.: model estimation.

## Data Availability

The original contributions presented in this study are included in the article. Further inquiries can be directed to the corresponding author.

## Appendix A. Stage Specifications

The system specifications are available at https://reurl.cc/vK9VXo (accessed on 21 June 2026).
